# The ontogeny of vocal identity in carrion crows (*Corvus corone*)

**DOI:** 10.1007/s10071-025-02021-5

**Published:** 2025-12-16

**Authors:** Hannah Gidl, Sara Binder, Anna N. Osiecka, Barbara C. Klump

**Affiliations:** 1 Department of Behavioral and Cognitive Biology, University of Vienna, Vienna, Austria; 2 Haidlhof Research Station, University of Vienna, Bad Vöslau, Austria; 3 Social, Cognitive and Affective Neuroscience Unit, Department of Cognition, Emotion, and Methods in Psychology, Faculty of Psychology, University of Vienna, Vienna, Austria; 4 Acoustics Research Institute, Austrian Academy of Sciences, Vienna, Austria; 5 ENES Bioacoustics Research Laboratory, University of Saint-Etienne, Saint- Etienne, France; 6 Institute for Theoretical Biology, Humboldt University of Berlin, Berlin, Germany; 7 Vienna Cognitive Science Hub, University of Vienna, Vienna, Austria; 8 Max Planck Institute of Animal Behavior, Radolfzell am Bodensee, Germany

**Keywords:** Corvids, Identity coding, Information theory, Vocal communication, Vocal development, Context-dependent vocalisations

## Abstract

For social species, the ability to identify individual group members is crucial. Vocalizations often carry individual signatures that can serve as cues for the caller’s identity. However, it is still unclear how consistent these signatures are and how early they develop. Here, we investigate the development of vocal individuality in hand-raised carrion crows. Over the course of four weeks, spanning both the nestling and fledgling stage, we recorded vocalisations of known individuals across various behavioural contexts. We show that vocal individuality is present early on and is encoded by several acoustic parameters whose importance remains consistent as the birds age. Additionally, while vocal dissimilarity between individuals generally increased over time, the pattern of change varied across contexts. Our findings highlight the dynamic nature of vocal individuality coding during early life and provide new insights into the relationship between vocal and social development in corvids.

**Supplementary Information:**

The online version contains supplementary material available at 10.1007/s10071-025-02021-5.

## Introduction

Acoustic communication plays an important role in animal interactions. In birds, vocal signals are a prominent aspect of communication (Catchpole et al. 2008) and individual differences in vocal signals can indicate the identity of the caller, often leading to individual vocal recognition (for a review, see Carlson et al. 2020). Recognizing individuals is of special relevance when interactions with the same individuals occur repeatedly over long periods of time (Dreiss et al. 2014). Examples range from female great tits (*Parus major*) being able to identify their partners’ songs (Lind et al. 1996) to song sparrows (*Melospiza melodia*) acoustically discriminating between familiar neighbours and strangers as well as between individual neighbours (Stoddard et al. 1991). Vocal recognition can also be important in family interactions: For example, in spectacled parrotlets (*Forpus conspicillatus*) siblings have been shown to recognize each other (Wanker et al. 1998), barn swallows (*Hirundo rustica*) may even adjust their competitive efforts according to relatedness with nestmates through acoustic kin recognition (Boncoraglio et al. 2009), and barn owls (*Tyto alba*) use individual vocal recognition to determine siblings’ motivation to compete for food brought to the nest by parents (Dreiss et al. 2014). Parent-offspring recognition is of special significance in colonially breeding species, where parents have to avoid misdirecting their care toward unrelated offspring (e.g. Levréro et al. 2009). In some species, parents can also discriminate between individual offspring, as has been found in black redstarts (*Phoenicurus ochruros*), where parents preferentially feed specific nestlings (Draganoiu et al. 2006).

In order for animals to discriminate between calls of different individuals, sufficiently distinct acoustic features need to be present in their vocalizations. Since the shape and size of the vocal tract and vocal apparatus shape a large part of the acoustic output, unique features likely exist in most vocalizing species due to slight morphological differences in each individuals’ vocal tract (Carlson et al. 2020). However, vocal individuality can also be affected by the social environment of an animal. Individuals may adjust the acoustic structure of their vocalisations to become more similar to social partners whose calls they hear frequently. For example, African penguins (*Spheniscus demersus*) seem to match their vocalisations to those of members of their colony (Baciadonna et al. 2022) and both little auks (*Alle alle*) and ravens (*Corvus corax*) show a tendency to match the structure of some call types to their partners’ (Luef et al. 2017; Osiecka et al. 2023).

Furthermore, growing evidence points towards the complex interactions between vocal identity and other important social cues, such as affective states. Across species, calls of negative valence tend to carry less identity information than those uttered in positive contexts (Osiecka *et al*. 2024a, Osiecka et al. 2024b). In mammals, high arousal also seems to partially override identity information (Volodin et al. 2017; Corvin et al. 2024), suggesting that vocal individuality may be more dynamic than previously thought.

Family signatures can be learned as early as during the embryonic stage, mitigating the risk of misdirected parental care toward brood parasites (Colombelli-Négrel et al. 2012). Individual vocal signatures may also change in their degree of inter-individual discriminability of calls through ontogeny, likely depending on the adaptive value of being recognizable. In red-crowned cranes (*Grus japonensis*), for example, the discriminability of the calls of the chicks increases from a first phase, in which the parents are territorial, to a second phase, in which the family transitions to flock living, a lifestyle change that leads to an increased risk of confusing ones’ own chicks with those of conspecifics (Klenova et al. 2009). However, to really disentangle what influences such ontological changes in vocal individuality, further studies on animals from different groups and life histories are needed.

Corvids represent an interesting model for studying aspects of communication, including acoustic communication (Suzuki and Izawa 2023; for a review, see Wascher and Reynolds 2025), as they live in complex societies with dominance hierarchies and long-term affiliative relationships and show highly complex cognitive skills (Taylor 2014; Bugnyar 2024).

Carrion crows (*Corvus corone*) are among the corvid species that are frequently used in social cognition research (e.g. Hillemann et al. 2014, Wascher 2015). They are known to have a complex and in some cases cooperative social life, with their breeding tactics ranging from territorial pair breeding to cooperative breeding (Baglione* et al.* 2002) and may therefore have both the ability and need for a complex system of vocal communication (Siriwardena 1995).

The social complexity of their societies suggests that they would benefit from individual vocal signatures and their recognition. Indeed, jungle crows (*Corvus macrorhynchos*) utilise so called “contact calls” for individual recognition of conspecifics (Kondo et al. 2010) and carrion crows are likely able to discriminate between alert calls from reliable and unreliable conspecifics (Wascher et al. 2015). Surprisingly however, there is little known about the ontogeny of vocal signatures in corvids.

Here, we recorded the vocalizations of five carrion crow chicks over a four-week period to investigate the ontogeny of vocal individuality during the nestling and fledgling stages. Specifically, we asked whether (a) calls can be classified to individuals and whether classification accuracy changes with age, (b) which acoustic parameters are important for encoding individuality and how these parameters vary with age and behavioural context, and (c) whether vocal dissimilarity between individuals changes with age as well as between context.

As adult carrion crows are able to recognize conspecifics by voice (e.g. Wascher et al. 2015), we expected that (a) calls can be classified to the individuals, but it was unclear whether vocal individuality would already be present at the nestling and fledgling stage. Early individuality might be advantageous for distinguishing between nestmates (e.g., during feeding), while the adaptive value of being recognizable by voice might increase post-fledging when the chicks start to intermingle with others. In accordance with findings from previous studies (e.g. Osiecka et al. 2024a), we predicted˙ that (b) the vocal individuality of calls would be carried by multiple acoustic parameters and that their relative importance might shift with age as the chicks’ vocalizations mature. Behavioral contexts might also influence the importance of individual parameters, as calls may vary structurally depending on the context in which they are produced, for example reflecting changes in affective state. Finally, we expected (c) vocal dissimilarity between individuals to increase with age, reflecting the need for clearer individual vocal signatures as social interactions become more complex. Behavioral contexts might also influence dissimilarity, because certain contexts could demand greater individuality in vocalizations (e.g., competitive *vs*. affiliative interactions).

## Materials and methods

### Study site and subjects

On May 1st 2024, five carrion crow (*Corvus corone*) nestlings were acquired from two wild nests in Lower Austria at an estimated age of 10–14 days. Carrion (all black) and hooded (partly gray) crows were previously considered two distinct species (Glandt 2012) with two stable hybrid zones, one of which runs through central Europe (De Knijff 2014). However, they have recently been combined and are currently considered subspecies of *Corvus corone* (AviList Core Team 2025). Therefore, and because our study subjects originate from one of the hybridization zones and thus include include both carrion (*Corvus corone corone*) and hooded (*Corvus corone cornix*) crows, as well as potential hybrids, we are not differentiating between phenotypes and refer to them as (carrion) crows throughout the manuscript.

The nestlings were individually marked for identification and hand-raised at Haidlhof Research Station near Bad Vöslau, Austria, following established procedures (see below) until fledging at 25 to 29 days of age. Sex of birds was unknown at the time of the study, but each bird was genetically sexed after data collection was completed (three females, two males). Following the STRANGE framework for animal behaviour research (Webster and Rutz 2020), we provide all known details on individuals in Table S1.

During the hand-raising period, nestlings were housed in two artificial nests indoors and in the same room. The nest properties were manipulated for a different experiment. One nest’s inside was lined with paper and contained two genetic siblings from the first wild nest. The other nest contained natural materials, such as sticks, straw and feathers, and consisted of two related siblings from the second wild nest as well as one unrelated social sibling from the first wild nest (Table S1). In the same room, three other crows that were not part of this study were hand-raised in one additional nest. Throughout, crows were exposed acoustically (through open windows) to vocalisations of the captive crow population (adults) at the Haidlhof Research Station as well as the local wild crow population. The crow nestlings were hand-fed every two hours from 6 am to 10 pm with a varied diet containing a mixture of ground fruit muesli, day-old chicks, mealworms, fruit, vegetables, feed lime, Korvimin, and fennel tea. They were weighed daily and if the weight gain rate was not as expected, we additionally provided Zophoba larvae.

After fledging, all five individuals moved to an outside aviary as a group, together with a sixth individual that was not hand-raised and therefore not included in this study. Crows were housed at the Haidlhof Research Station in the acoustic vicinity of the captive crow population and both visually and acoustically exposed to the local wild crow population. Food and water were provided *ad libitum*. The crows’ daily diet consisted of fruit muesli, seeds, fresh fruits or vegetables, and meat, with adjustments throughout the week including day-old chicks, eggs, and supplements such as Korvimin, feed lime, garlic powder, and herb blends.

## Data collection

Audio recordings were collected in stereo on a handheld recorder (Sennheiser Zoom H4n Pro, sampling rate 48 kHz, amplitude resolution 16 bits) attached to a directional microphone (Sennheiser MKE 600) mounted on a rubber grip with a vibration decoupler. An additional mini-microphone was attached to the second channel of the recorder for annotation purposes. During recording sessions (see below), after a bird vocalized, we recorded the identity of the vocalising bird and the behavioural context in which the vocalisation occurred on this second channel (for a definition of the behavioural contexts see Table S2).

Audio recordings took place over two time periods: (1) during the hand-raising period (Phase 1, i.e. the nestling stage, start: 11–14 days of age, duration: 15 days), and (2) afterwards in an outside aviary (Phase 2, i.e. after fledging, start: 25–29 days of age, duration: 14 days).

During Phase 1, the birds were recorded daily, every two hours between 6 am and 6 pm. Each recording session consisted of recordings of: (i) 5 min before the start of feeding, (ii) the duration of the feeding session (mean: 5 min 4 s, range: 1 min 22 s to 12 min 34 s) and (iii) 5 min after feeding. The birds remained in their nests during the recording and recording sessions were assigned to nests in a pseudo-randomised order, making sure that each nest was featured a similar amount for each of the feeding sessions, as well as in am and pm recordings each week. Note, however, that not all recordings were included in the analysis (for details on the annotation and selection process see below).

In Phase 2, the birds were recorded three times a week. Each recording session consisted of (i) 5 min *ad libitum* recording, (ii) 5 min focal recording of each individual, the order was randomised within sessions, (iii) 5 min *ad libitum* recording. Recording sessions were performed am or pm in a pseudo-randomised order (i.e. the ratio of am and pm recordings did not exceed 1:2 and alternated each week).

## Audio analysis

All analyses were performed in R v. 4.4.2 (R Core Team 2024).

The obtained audio material was screened manually in Raven Lite version 2.0.5 (K. Lisa Yang 2024) (Phase 1 by HG, Phase 2 by SB) to identify and extract good quality (i.e. non-overlapping, good signal-to-noise ratio) calls for each individual. For Phase 1, at least one recording of each nest was analysed per recording day. For this, we selected the closest available recording to 12 pm, as birds were observed to vocalise less in the morning and evening. Where two recordings with the same distance to 12 pm were available, we selected the later one. For seven of the 15 days in Phase 1, instead of annotating only one, two to four recordings were annotated for a given nest to obtain a sufficient number of calls for all individuals. Extracted calls were cut to the exact start and end of the audio signal using the *call.detect* function of the *callsync* package (Smeele et al. 2024). We decided to exclude calls in the *fed* context (see Table S2), as the size and type of food passing through the esophagus might have influenced the acoustic features of these vocalisations. The final dataset consisted of 2221 calls (for number of calls per individual, context and age class, see Supplementary Material). Cut files were batch analysed using the *analyze* function of the *soundgen* package (Anikin 2019, pitch floor of 400 Hz, pitch ceiling of 6000 Hz, 1 ms step) to extract a set of spectral descriptors of the signal. We selected eight parameters relevant to identity coding across species that were successfully extracted for all calls (see Table 1).

**Table 1:** Acoustic parameters extracted for all calls. Definitions as per the *analyze* function manual (Anikin 2019)

Parameter	Definition
Duration (s)	Duration of the vocalisation
Mean amplitude	Mean root mean square of amplitude per frame
Peak frequency (hz)	The frequency with maximum spectral power
25th quartile (hz)	The 25th energy quantile of the spectrum
50th quartile (hz)	The 50th energy quantile of the spectrum
75th quartile (hz)	The 75th energy quantile of the spectrum
Spectral centroid (hz)	The center of gravity of the frame’s spectrum
Spectral slope (db/khz)	The slope of linear regression fit to the spectrum

### Data analysis

To decide whether the data should be used raw or in reduced dimensions, we checked its suitability for factor analysis using the *KMO* function of *EFAtools* package (Steiner and Grieder 2020). With an overall KMO score of 0.645 the suitability was mediocre, and we decided to use raw acoustic parameters as input values for further models.

## a) Classification to individual

First, we used permuted discriminant function analysis (pDFA, Mundry and Sommer 2007) to see whether calls could be assigned to individuals within and across age classes. To do this, we ran six models in total, including five models grouping the animals into seven-day age classes (i.e., 11–17, 18–24, 25–31, 32–38, and 39–45 days after the estimated hatching date), and one model pooling all age classes together. Each model used the *pDFA.nested* function (pDFA script provided by R. Mundry, based on the *MASS* package, Ripley et al. 2013) with behavioural condition as the restriction factor and 1000 permutations.

### b) Parameters coding identity

To assess which acoustic parameters have the biggest potential to carry cues for caller identity, we calculated the potential of identity coding (PIC, Robisson 1992) using the *calcPIC* function of the *IDmeasurer* package (Linhart 2019). Additionally, we used the output values in a simple linear model (*lm* function, *stats* package) to check whether any PIC changes occured with age. This was performed for (1) all contexts pooled together, and (2) for “beg”, “rest”, “touch-affiliative” and “touch-aversive” contexts separately. To account for multiple testing, we used Bonferroni adjustment to the p-value, which means that significance was retained at *p* = 0.006.

## c) Ontogeny of vocal dissimilarity

To investigate whether and how vocal dissimilarity between individuals might change with age, we first calculated median values for each acoustic parameter for each individual and day. We then calculated the Euclidean distance matrix (*dist* function, *stats* package) for each dyad per day. Dissimilarity was then calculated as the difference between the maximum distance and the obtained distance for each dyad in the matrix. A dissimilarity score of 0 indicates perfect similarity (self-correlation) with larger values indicating higher dissimilarity. These values were then used in a linear model (*lm* function, *stats* package) with the dissimilarity score as a response and age in days as a predictor variable. This was performed for (1) all behavioural contexts pooled together, and for (2) each behavioural context separately: that is, we looked at whether *any* sounds produced by each dyad differentiated more with age, and whether the vocal dissimilarity *within each context* changed with age. With Bonferroni adjustment, significance was retained at *p* = 0.007.

## Results

**Fig. 1:**
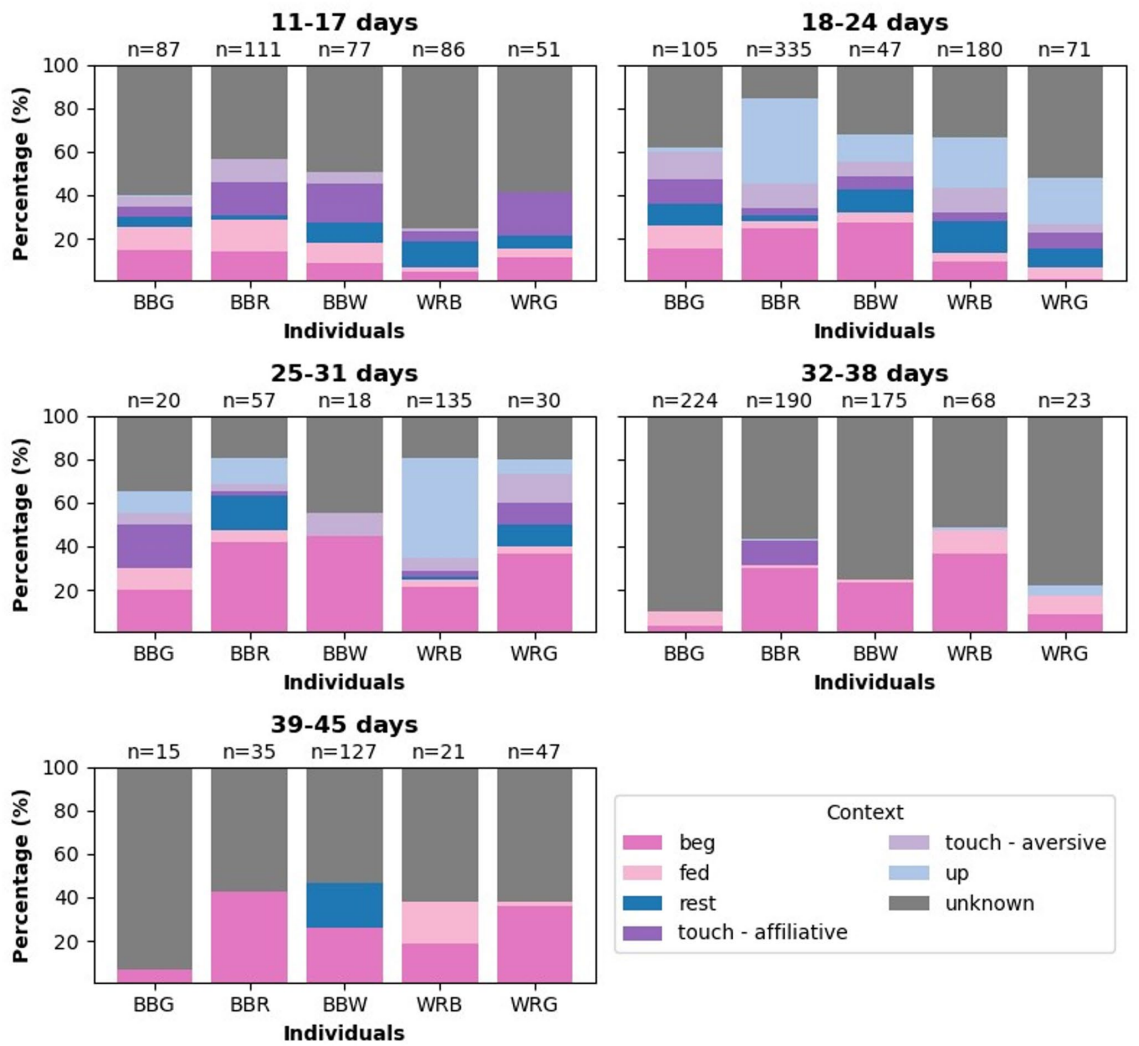
Contexts in which calls occurred for all individuals and age periods

The composition of calls of various contexts differed between individuals and changed with age (Fig. 1, see Figure S1 for examples of calls of different contexts and Figure S2 of change of begging calls across age classes).

### Classification to individual

Calls could be assigned to the individual within and across age classes significantly above chance levels (Table 2) and there was no apparent pattern in the efficiency of this classification with age.

**Table 2:** Results of the permuted discriminant function analysis for each age class and all ages pooled together

	11–17 days	18–24 days	25–31 days	32–38 days	39–45 days	Pooled
No. context categories	9	11	10	4	5	12
Total no. calls	376	703	250	652	240	2221
Correctly classified (%)	47.91	50.79	60.88	61.11	59.68	41.12
Chance level (%)	30.11	31.81	36.00	39.24	31.79	25.34
*p* value for classified	**0.001**	**0.001**	**0.001**	**0.001**	**0.002**	**0.001**
Correctly cross-classified (%)	44.85	48.89	60.68	50.65	39.29	46.08
Chance level for cross-classified (%)	19.93	25.45	25.28	20.99	24.91	22.37
Relative cross-classification level	2.25	1.92	2.40	2.41	1.58	2.06
*p* value for cross-classified	**0.001**	**0.001**	**0.001**	**0.001**	**0.002**	**0.001**

### Parameters coding identity

All parameters showed PIC values close to 1, i.e. the usual cut-off for importance to identity coding (Robisson* et al.* 1993). However, none of them showed particularly high values (i.e. over 2, Table 3). Parameters’ importance across contexts did not change with age (Table 3, Supplementary Material). For the affiliative touch context, the importance of the 50th frequency quantile and amplitude tended to decrease with age (Table S6), but this was not significant after Bonferroni correction.

**Table 3:** Potential of identity coding of the eight acoustic parameters across age classes. Values over 1 indicate importance for identity coding

Parameter	11–17 days	18–24 days	25–31 days	32–38 days	39–45 days	*p*-value
Duration (s)	1.13	1.12	1.31	1.07	1.17	0.896
Mean amplitude	1.00	1.01	1.20	1.10	1.27	0.076
Peak frequency (hz)	1.03	0.97	1.01	0.98	1.22	0.269
25th quartile (hz)	1.07	0.98	1.01	0.98	1.25	0.391
50th quartile (hz)	1.04	1.07	1.01	1.07	1.19	0.196
75th quartile (hz)	1.03	1.10	1.04	1.04	1.31	0.220
Spectral centroid (hz)	1.03	1.10	1.02	1.09	1.24	0.157
Spectral slope (db/khz)	1.01	1.05	1.13	1.97	1.21	0.369

### Ontogeny of vocal dissimilarity

**Fig. 2:**
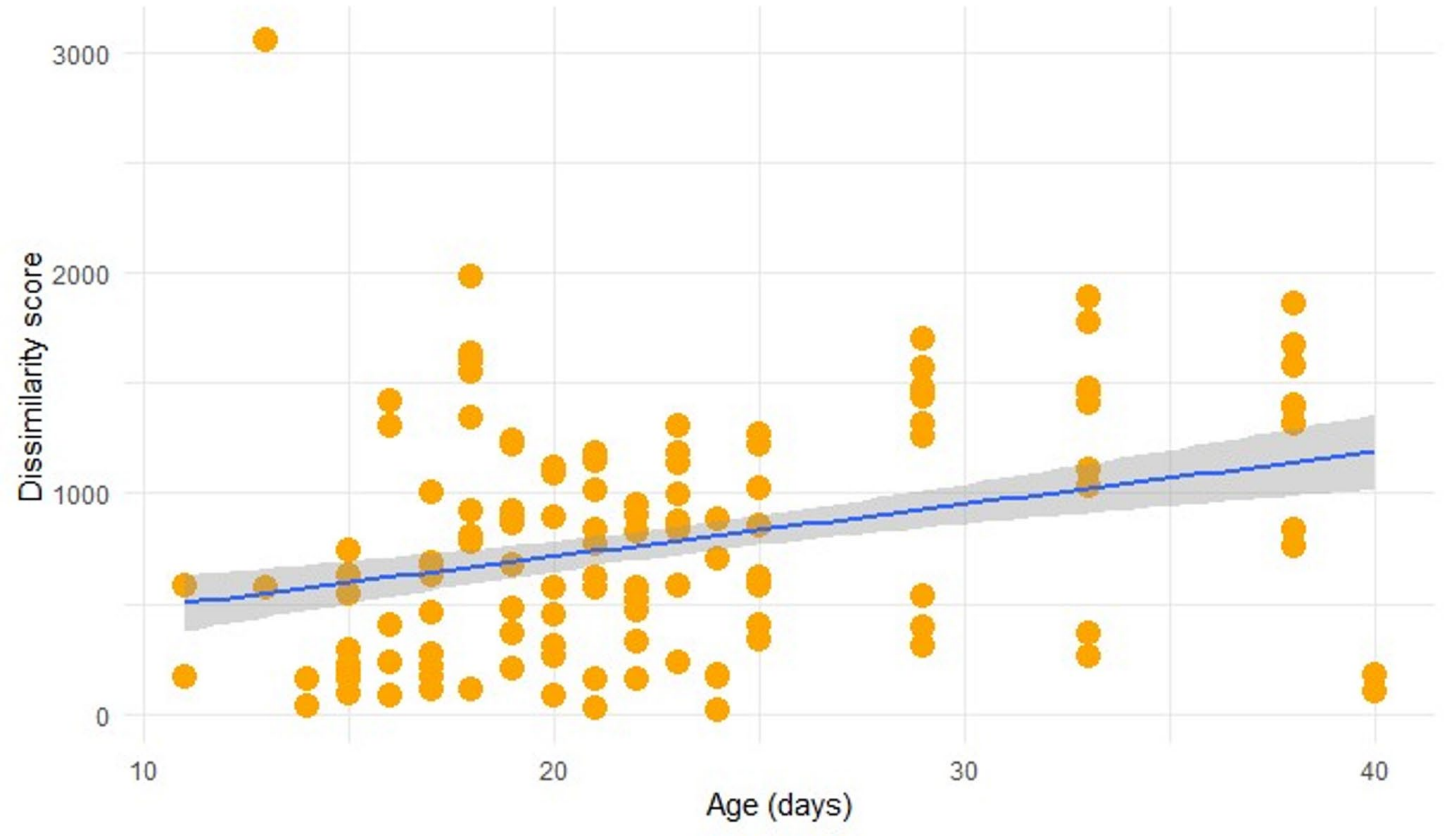
Linear model of the overall vocal dissimilarity between each two birds with age. All behavioural contexts are pooled together

**Fig. 3:**
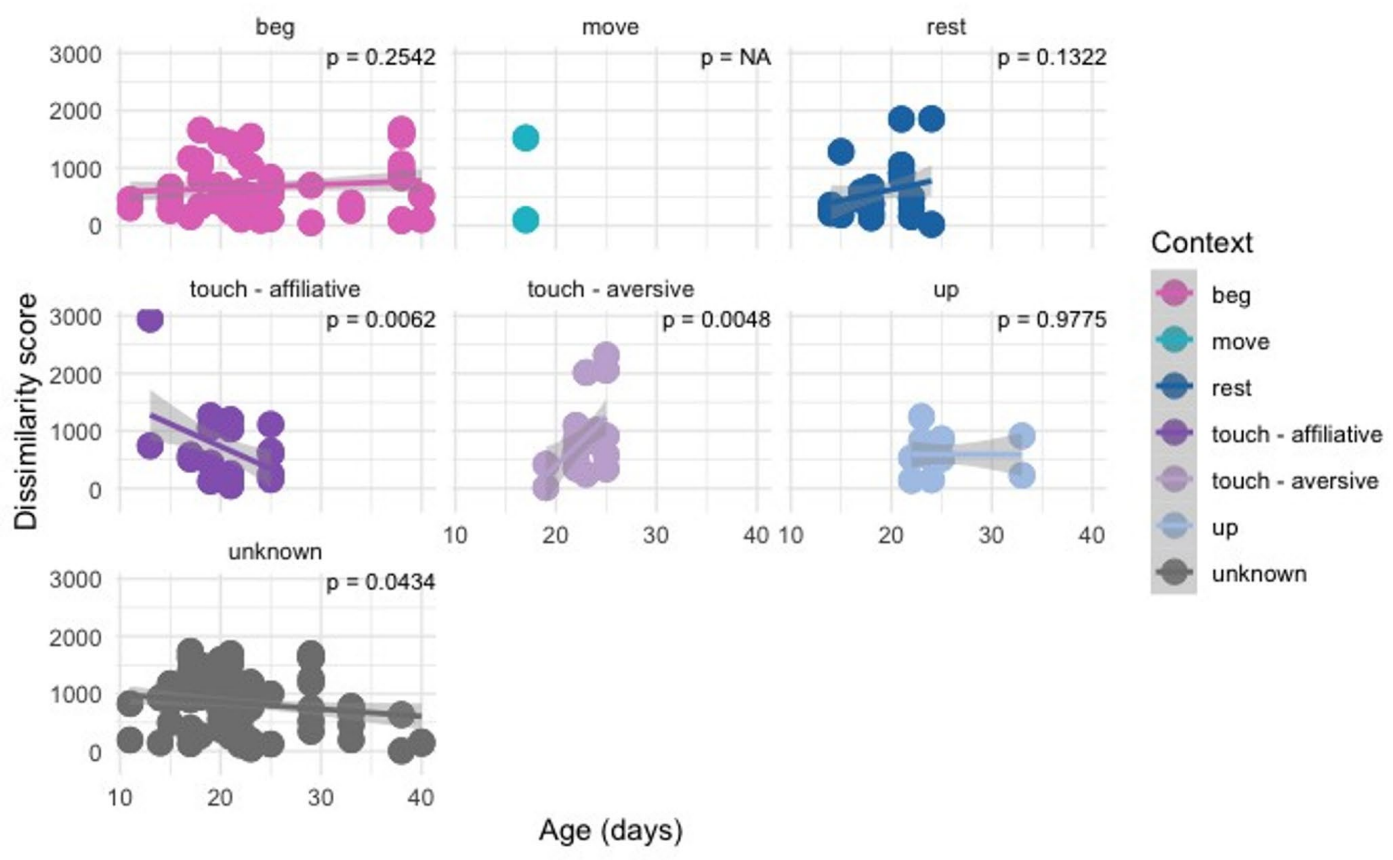
Linear models of the vocal dissimilarity between each two birds in each behavioural condition

The overall vocal dissimilarity between the birds (i.e., not taking into account the behavioural context of vocalisation) increased with age (*p* < 0.0001, Fig. 2). There was a significant increase in vocal dissimilarity with age in the “touch-aversive” context (*p* = 0.0048), decrease in the “touch-affiliative” context (*p* = 0.0062) and a tendency to decrease in the pooled “unknown” context (*p* = 0.0434), Fig. 3). Data for the “move” category were insufficient for statistical analyses.

## Discussion

This study provides, to our knowledge, the first description of the ontogeny of vocal individuality across behavioural contexts in a corvid species, namely the carrion crow. We found that calls could be correctly assigned to individuals above chance level, both within and across age classes, indicating that vocal individuality is present from early on. This identity was encoded by multiple acoustic parameters throughout the study period, demonstrating that vocal individuality was based on a combination of acoustic parameters. Additionally, across contexts, vocal dissimilarity between individuals increased with age, suggesting that vocal individuality becomes more pronounced throughout the development from nestlings to fledglings. However, how vocally different individuals were from each other and how this dissimilarity changed over time varied strongly across behavioural contexts, highlighting the dynamic nature of vocal individuality and its relationship with the social functions of the calls.

To put our results in a broader context, it should be noted that they are based on a small sample of five hand-raised individuals and as such, their generalisability should be treated with some caution. While future studies with a larger sample size are needed to fully understand the vocal development in the species, this is the first time that the vocal development of individual corvid chicks has been followed from such an early age. The presence and development of vocal identity - despite the small sample size - represents therefore an important step in broadening our understanding of the intersection between behavioural context and ontogeny of vocal individuality. The raising environment could also have influenced the crows’ vocal development, and hence our results, due to the exposure to human caregivers and mainly same-age individuals. This setting - while necessary to obtain high-quality vocal data of known individuals - does not fully reflect natural conditions, where parent-offspring vocal interactions may play an important role in vocal development. We limited these potential drawbacks by taking the chicks from wild nests at 10–14 days old, thereby exposing them to parental and local vocal input early on and vocally exposing them to both same-age and adult crow vocalisations from both the captive and local wild crow populations at the Research Station throughout the study period. We found vocal identity to be present from early on and did not observe any sounds reminiscent of human vocalisations, suggesting that the presence and development of vocal identity was robust to our experimental set-up.

Crow calls in our dataset were individual (i.e. could be assigned to the correct individual above chance level) from early on and in all five age classes included in the study period (from 11 to 45 days of age). This aligns with previous research in young passerine birds of other species that found that vocal individuality was present from an early age. For example, in Spanish sparrow (*Passer hispaniolensis*) nestlings, calls could be classified to individuals before the age of 10 days (Marques et al. 2011) and in zebra finches (*Taeniopygia guttata*), begging calls were highly individual well before fledging (Levréro et al. 2009). While we can currently not exclude the possibility that vocal individuality in corvids develops within the first 10 days after hatching, the early presence of vocal individuality in crows may serve several adaptive functions: one possibility is that parents use individual vocal cues to preferentially allocate food to specific nestlings (e.g. Draganoiu *et al*. 2006). Additionally, nestlings might benefit from recognizing their siblings’ calls, as this could facilitate vocal interactions within the brood. This has been observed in barn owls, where vocal individuality plays a role in sibling negotiations (Dreiss et al. 2014). If similar mechanisms exist in crows, early vocal individuality may be as relevant in early life as later in development.

The obtained Potential of Individuality Coding (PIC) values suggest that - as expected - multiple parameters contribute to vocal identity coding in crows. These results align with previous studies demonstrating that identity information is often encoded across multiple acoustic features rather than being conveyed by a single dominant parameter. For example, in zebra finch nestlings (Levréro et al. 2009) and macaroni penguins (*Eudyptes chrysolophus*), individual identity is encoded through both temporal and spectral characteristics (Searby et al. 2004; for further examples see e.g. Searby and Jouventin 2005, Jouventin and Aubin 2002). As crows live in complex social environments, they likely need a robust encoding system including parameters that span different acoustic domains, so that identity cues are less susceptible to degradation during transmission, as proposed by (Osiecka et al. 2024c, d). Encoding identity across multiple acoustic features may thus enhance the reliability of identity recognition across different social and physical environments. It is, however, important to note that crow calls are very chaotic, with a strong presence of non-linear phenomena, and without a strong fundamental frequency outline. It is thus understandable that the individual differences arose from an overall shape of the call, rather than a “signature style” frequency outline. Interestingly, and against our predictions, neither when pooling across contexts, nor when looking within contexts, did we find significant changes in parameter importance with age. This suggests that the acoustic structure used for identity coding remains largely consistent across contexts and over time as the individuals grow, possibly facilitating long-term social recognition.

While crow calls could be assigned to the correct individual above chance level across all age classes, showing vocal individuality from early on, the analysis of vocal dissimilarity scores suggests that this individuality becomes more pronounced as crows age. This may be due to morphological changes that occur with growing, but an alternative explanation is that increased cognitive and motor control enables more consistent vocal production, as suggested for passerines (Marques* et al.* 2011). Additionally, environmental and social factors may drive this change: As young crows interact more with others, the need to be recognizable by voice may become stronger (Klenova et al. 2009). Interestingly, when analyzing dissimilarity scores separately in different contexts, we found that vocal dissimilarity increased in the aversive touch context (i.e. being cleaned by a human), while it decreased in the affiliative touch context (i.e. being touched by a human to initialize feeding). This contrasts with previous findings in birds and mammals, where calls showed lower individuality scores in aversive than affiliative (e.g. little auks, *Alle alle*, Osiecka et al. 2024a) and rhinos, *Ceratotherium simum simum*, Linn et al. 2021), and in negative than positive contexts (Osiecka et al. 2024b). Further research on a larger number of birds is now needed to further investigate the possible function of decreased individual signalling in affiliative contexts.

Our study focuses on whether acoustic differences encoding identity could be detected. To fully chart the importance of vocal identity in this species, further studies are now needed to address the question of how the birds themselves perceive these differences (e.g. through playback studies) and in which way they facilitate recognition by conspecifics (e.g. discrimination experiments).

## Conclusion

To our knowledge, our study is the first to examine how individuality changes over time across different contexts in the corvid family. We show that while crow calls are individually distinctive from early on, they do become overall more dissimilar with age. The level of dissimilarity between individuals depends on the behavioural context, and thus the social function of that call. This highlights the complex nature of vocal individuality, and points towards the importance of correcting for the social functions of the studied utterances when individuality is considered.

## Supplementary Information

Below is the link to the electronic supplementary material.

Supplementary Material 1

## Data Availability

Full codes generated in this study are available at https://osf.io/md7cf/?view_only=b94592d7fbf3492387f7df06aa4f0d87 and can be reused with appropriate citation.
