# Supplementary material for: The ontogeny of vocal identity in carrion crows (Corvus corone)
Source: Anim Cogn. 2025 Dec 16;29(1):17. doi: 10.1007/s10071-025-02021-5 (PMC12852247; doi:10.1007/s10071-025-02021-5)
Supplement: Supplementary file 1 — Supplementary Material 1 [file 10071_2025_2021_MOESM1_ESM.pdf]

Supplementary Material

Manuscript title: The ontogeny of vocal identity in carrion crows *Corvus corone*

Authors: Hannah Gidl, Sara Binder, Anna N Osiecka, Barbara C. Klump

Journal: Animal Cognition

Figures

- **Figure S1**: Calls produced in three behavioural contexts (begging, resting, and touching) by three chicks of roughly the same age ($\tilde{2}$5 days after hatching)
- **Figure S2**: Begging calls produced by three chicks in five age classes.

Tables

- **Table S1**: Data on individuals
- **Table S2**: Behavioral contexts and definitions used during audio recordings
- **Table S3**: Number of calls per age class in final dataset
- **Table S4**: Potential of identity coding across age classes in the 'rest' context
- **Table S5**: Potential of identity coding across age classes in the 'beg' context
- **Table S6**: Potential of identity coding across age classes in the 'touch - affiliative' context
- **Table S7**: Potential of identity coding across age classes in the 'touch - aversive' context

1 Supplementary Figures

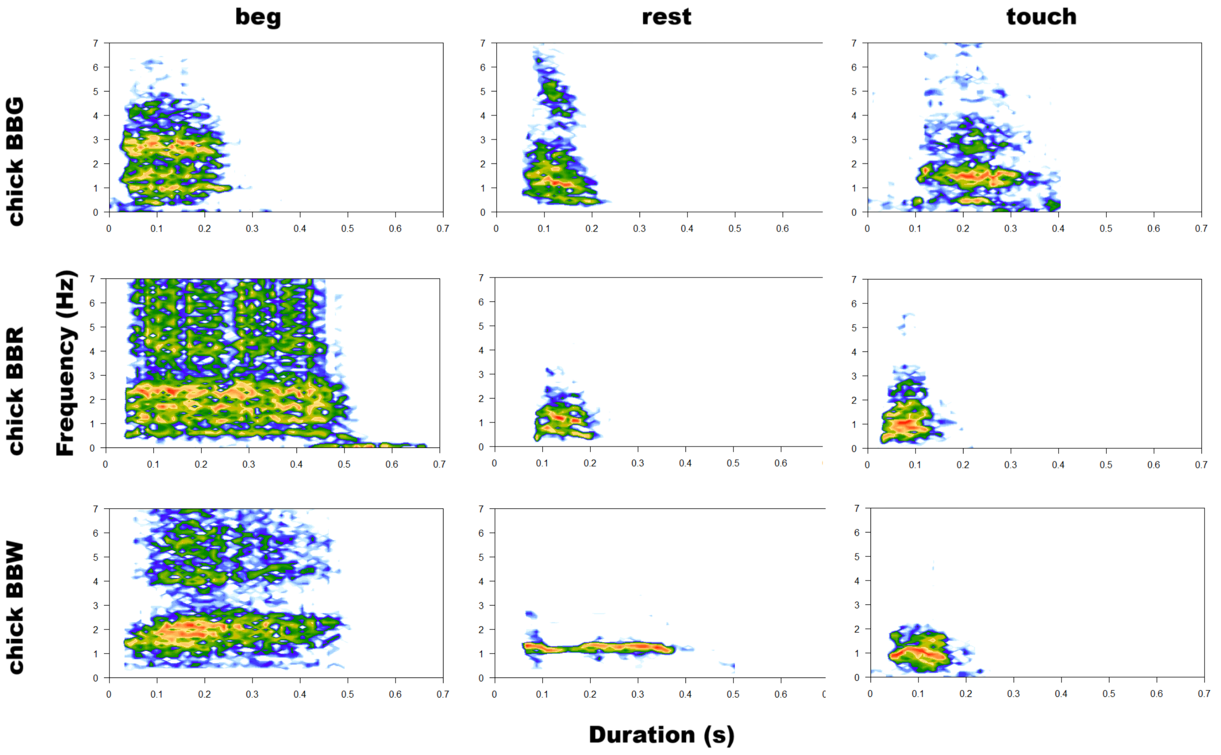

Figure S1: Calls produced in three behavioural contexts (begging, resting, and touching) by three chicks of roughly the same age (2̃5 days after hatching)

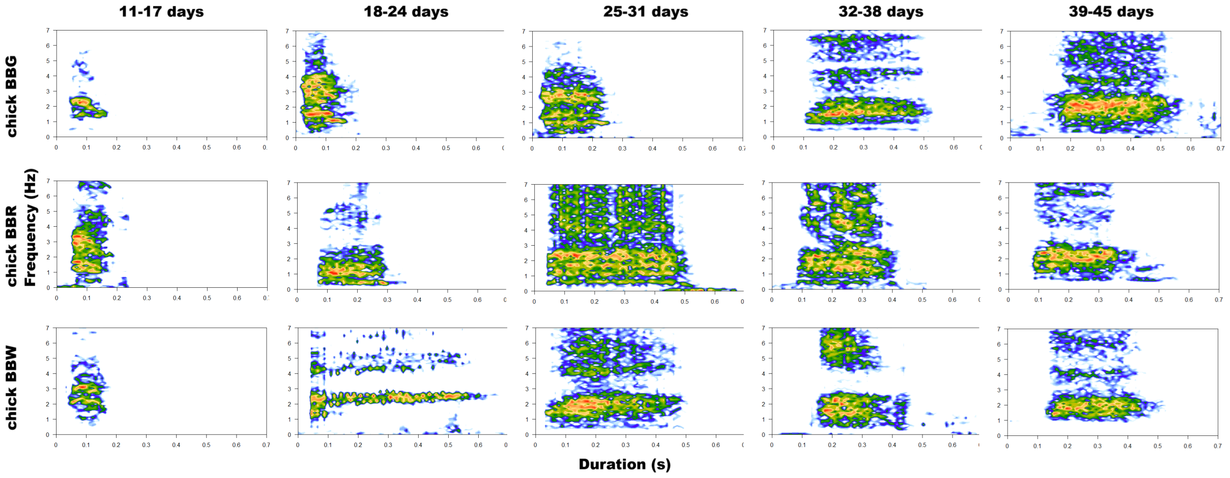

Figure S2: Begging calls produced by three chicks in five age classes. BBG and BBR have a low vocal dissimilarity (48.48). BBG and BBW have a high vocal dissimilarity (1671.08)

2 Supplementary Tables

Table S1: Data on individuals including estimated hatching dates, estimated age at the start of phases 1 and 2, original and artificial nest numbers, nest types, sex, and no of calls in the final dataset.

individual	estimated hatching date	age in days/phase 1	age in days/phase 2	original nest	artificial nest	nest type	sex	no. of calls in final dataset
BBR	2024-04-21	10	25	MA1	1	natural	m	695
BBG	2024-04-21	10	25	MA1	1	natural	m	415
BBW	2024-04-21	10	25	MA1	2	paper	f	433
WRG	2024-04-17	14	29	AN1	2	paper	f	212
WRB	2024-04-17	14	29	AN1	2	paper	f	466

Table S2: Behavioural contexts and definitions used during audio recordings.

behavioural context	definition	no. of calls in final dataset
beg	beak is open wide, head tilted up, in some cases flapping wings	455
up	standing / sitting up tall, neck extended	273
fed	receiving a piece of food by a caretaker	excluded from analyses
touch - aversive	being touched on beak or neck by caretaker in an aversive context (usually being cleaned)	115
touch - affiliative	being touched on beak or neck by caretaker in an affiliative context (usually to initialize feeding)	121
move	caretaker is moving or tapping the nest	17
disturb	nestmate moves around, resulting in the vocalisation	32
social	allopreening or bill twining	1
rest	sitting/crouching/laying down, eyes are open	122
sleep	crouching/laying down, eyes are closed or head is tucked into feathers	8
comfort	autopreening or scratching	2
flight	flight training; flapping of wings two or more times	4
unknown	none of the pre-defined behavioural contexts was recorded for the given vocalisation	1071

Table S3: Number of calls per age class in final dataset

age class	no. of calls in final dataset
11-17 days	376
18-24 days	703
25-31 days	250
32-38 days	652
39-45 days	240

Table S4: Potential of identity coding of the eight acoustic parameters across age classes in the "rest" context. Values over 1 indicate importance for identity coding. There were insufficient data to calculate the values for the 32-38 age class.

parameter	11-17 days	18-24 days	25-31 days	32-38 days	39-45 days	p-value
duration (s)	1.22	1.11	2.29	NA	1.00	0.955
mean amplitude	1.05	1.33	1.09	NA	1.00	0.591
peak frequency (hz)	0.84	1.07	2.17	NA	1.00	0.835
25th quartile (hz)	0.93	1.05	1.50	NA	1.00	0.832
50th quartile (hz)	1.12	1.08	1.11	NA	1.00	0.110
75th quartile (hz)	0.93	1.10	1.33	NA	1.00	0.859
spectral centroid (hz)	1.11	1.18	1.27	NA	1.00	0.951
spectral slope (db/khz)	1.05	1.10	1.25	NA	1.00	0.986

Table S5: Potential of identity coding of the eight acoustic parameters across age classes in the "beg" context. Values over 1 indicate importance for identity coding.

parameter	11-17 days	18-24 days	25-31 days	32-38 days	39-45 days	p-value
duration (s)	1.25	1.21	1.93	1.75	1.13	0.820
mean amplitude	0.98	0.87	1.01	1.52	1.25	0.173
peak frequency (hz)	1.33	1.13	0.99	1.23	1.25	0.891
25th quartile (hz)	1.22	1.10	1.08	1.25	1.26	0.489
50th quartile (hz)	1.24	1.16	1.12	1.45	1.01	0.797
75th quartile (hz)	1.17	1.22	1.48	1.32	1.14	0.957
spectral centroid (hz)	1.26	1.19	1.34	1.29	1.11	0.558
spectral slope (db/khz)	1.10	1.03	1.32	1.18	1.18	0.434

Table S6: Potential of identity coding of the eight acoustic parameters across age classes in the "touch - affiliative" context. Values over 1 indicate importance for identity coding. There were insufficient data to calculate the values for the '32-38 days' and '39-45 days' age classes.

parameter	11-17 days	18-24 days	25-31 days	32-38 days	39-45 days	p-value
75th quartile (hz)	1.74	1.65	0.93	NA	NA	0.106
50th quartile (hz)	1.97	1.59	1.11	NA	NA	0.025
25th quartile (hz)	1.42	1.08	1.71	NA	NA	0.759
duration (s)	1.04	1.90	3.36	NA	NA	0.845
mean amplitude	1.29	1.24	1.12	NA	NA	0.015
peak frequency (hz)	1.32	1.11	2.89	NA	NA	0.882
spectral centroid (hz)	1.80	1.63	0.91	NA	NA	0.096
spectral slope (db/khz)	1.33	1.02	1.11	NA	NA	0.235

Table S7: Potential of identity coding of the eight acoustic parameters across age classes in the "touch - aversive" context. Values over 1 indicate importance for identity coding. There were insufficient data to calculate the values for the '32-38 days' and '39-45 days' age classes.

parameter	11-17 days	18-24 days	25-31 days	32-38 days	39-45 days	p-value
75th quartile (hz)	1.191	1.397	3.500	NA	NA	0.282
50th quartile (hz)	1.238	1.299	2.970	NA	NA	0.314
25th quartile (hz)	1.701	1.201	2.547	NA	NA	0.573
duration (s)	1.175	1.782	1.131	NA	NA	0.962
mean amplitude	1.056	1.413	2.346	NA	NA	0.160
peak frequency (hz)	1.426	1.272	2.038	NA	NA	0.455
spectral centroid (hz)	1.136	1.362	3.077	NA	NA	0.265
spectral slope (db/khz)	1.203	1.359	1.294	NA	NA	0.605
